# Study on the Ratio and Model Test of Similar Materials of Heavily Weathered Granite

**DOI:** 10.3390/ma17215324

**Published:** 2024-10-31

**Authors:** Guofeng Hu, Weihao Song, Xinran Yu, Mingbao Lin, Yunlong Tie, Ben He

**Affiliations:** 1 Dongtai Double Innovation Energy Development Corporation Limited, Yancheng 224237, China; 2 School of Civil Engineering, Shandong University, Jinan 250061, China; 3 Key Laboratory of Far-Shore Wind Power Technology, Power China Huadong Engineering Corporation Limited—HDEC, Hangzhou 311122, China

**Keywords:** similar material ratio test, model test, large single pile, offshore foundations

## Abstract

To study the bearing characteristics of rock-socketed single piles on the southeast coast of Fujian Province, we conducted similar material ratio tests and single pile model tests. Initially, based on the mechanical parameters of strongly weathered granite, 10 groups of similar material samples were prepared using iron concentrate powder, barite powder, and quartz sand as aggregates, with rosin and alcohol as the cementing agents and gypsum as the modulating agent. Through triaxial testing and range and variance analysis, it was determined that the binder concentration has the most significant impact on the material properties. Consequently, Specimen 1 was selected as the simulation material. In the model test, the strongly weathered granite stratum was simulated using the ratio of Specimen 1. A horizontal load was applied using a pulley weight system, and the displacement at the top of the pile was measured with a laser displacement meter, resulting in a horizontal load–displacement curve. The results indicated that the pile foundation remained in an elastic state until a displacement of 2.5 mm. Measurements of the horizontal displacement and bending moment of the pile revealed that the model pile behaves as a flexible pile; the bending moment initially increases along the pile length and then decreases, approaching zero at the pile’s bottom. The vertical load test analyzed the relationship between vertical load and settlement of the single pile, as well as its variation patterns. This study provides an experimental basis for the design of single pile foundations in weathered granite formations on the southeast coast of Fujian Province and aids in optimizing offshore wind power engineering practices.

## 1. Introduction

The development and utilization of clean energy has become an important strategic direction of global energy development. In this context, offshore wind energy has become one of the new energy sources vigorously developed due to its advantages of stable wind speed, strong wind power, and small environmental impact [1,2]. In the development of offshore wind power, the design and stability of the wind power base are of paramount importance. Due to the complexity of the offshore environment, the foundation needs to bear a variety of loads [3,4,5]. Reasonable selection and design of the foundation form can not only ensure the normal operation of the fan, but also reduce the overall cost of the wind power project. At present, the foundation forms of offshore wind power mainly include single pile foundation, gravity foundation, tripod foundation, jacket foundation, and floating foundation [6,7,8].

In order to ensure the safety and reliability of wind power foundation, a similar material ratio test has been widely used in geotechnical engineering [6,9]. The similar materials can simulate the engineering geological conditions of the site well and provide a solid foundation for the follow-up study. At present, many scholars have conducted a lot of research in this field. Chen et al. [10] studied the ratio and mechanical properties of low-strength similar materials, which are suitable for the physical simulation of coal mining and related geotechnical engineering. Jin-y [11] studied the ratio of similar materials in physical model tests by uniform design method, and analyzed the physical and mechanical properties of materials. Zhang et al. [9] developed a new type of similar material for foundation pit excavation model test, and studied the effects of various raw materials on the properties of similar materials. Zong-pa [12] studied the ratio of similar materials in the geomechanical model and emphasized the influence of binder concentration on material properties. Liu and Liu [13] developed a new type of fluid–solid coupling similar material, studied the properties of the material at different ratios, and analyzed the relationship between the parameters and the material composition.

Through model test and numerical simulation, the deformation mechanism of pile foundation under horizontal load, vertical load, and bending moment has been deeply studied. Ma et al. [14] used FLAC3D to analyze the horizontal load single pile shear force, bending moment, p–y curve, and other parameters. Dong et al. [15] analyzed the influence of boundary effect of model box on the horizontal bearing capacity of pile foundation through finite element method, and proposed a formula to predict the influence of boundary effect on the difference of horizontal bearing capacity of uniform clay. Arshad and O‘Kelly [16] conducted a comprehensive analysis of the response of single pile foundation under various loads, pointing out the limitations of the existing model and suggesting a direction for future research. Kim et al. [7] and Klinkvort et al. [17] focused on the behavior of a single pile under horizontal load, and analyzed the applicability of existing design specifications and the uncertainty caused by scaling effect. Trojnar [18] proposed the design method of hybrid single pile foundation and demonstrated its advantages in reducing horizontal displacement. Liu and Dai [19] further enriched understanding of the behavior of pile foundation under complex loads by studying the deformation and failure mechanism of rock under cyclic loading.

The objective of this study is to investigate the influence of similar material ratios on the bearing performance of single pile foundations within the strongly weathered granite stratum. By conducting systematic tests, we aim to assess how varying material compositions affect the deformation and bearing capacity of these foundations under different load conditions. This research will provide essential insights to optimize the engineering design of single pile foundations for offshore wind power projects, ultimately supporting advancements in offshore wind energy development.

## 2. Similar Material Ratio Test Scheme

As a key technology in geotechnical mechanical model tests, the ratio of similar materials plays an irreplaceable role in simulating the physical and mechanical properties of various rocks and soils [20,21,22]. The selection and ratio of materials directly affect the accuracy of the simulation effect and the reliability of the experimental results [23,24,25]. Based on the analysis of previous research [26,27,28,29,30], we selected iron concentrate powder, barite powder, and quartz sand as aggregates due to their high strength and ability to replicate the mechanical properties of granite. Rosin and alcohol were used as the cementing agents, ensuring the mixture maintains structural integrity, while gypsum was chosen as the regulating agent to adjust cohesion and internal friction. According to the characteristics of the heavily weathered granite layer in the coastal area of Fujian Province, the corresponding similar material ratio scheme was designed with the elastic modulus, cohesion force, and internal friction angle as the main control parameters, and the combination of materials and the ratio were optimized to simulate the mechanical properties of the heavily weathered granite in this area as true as possible.

**Figure 1:**
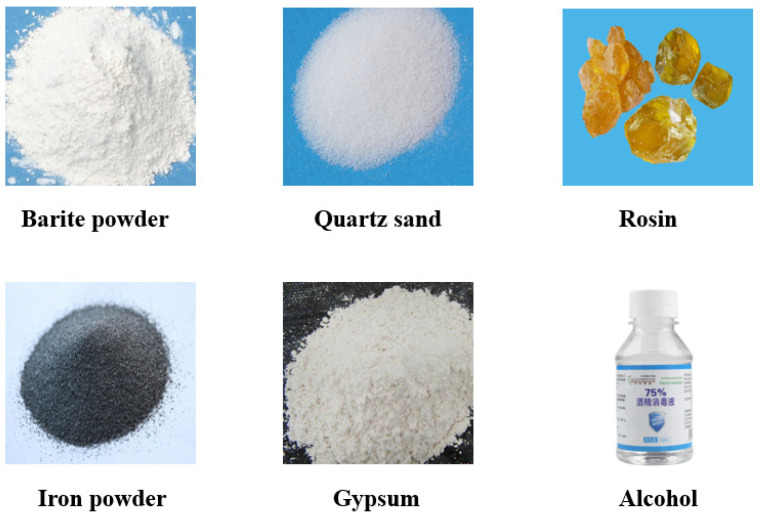
Raw materials of similar materials.

Figure 1 shows the appearance of the raw materials. In the similar material model test, we set up four main factors to investigate the influence of different material ratios on the simulation effect: factor A: quality of iron concentrate powder/(iron concentrate powder + barite powder + quartz sand), factor B: quality of barite powder/(iron concentrate powder + barite powder + quartz sand), factor C: (gypsum and rosin)/(iron concentrate powder + barite powder + quartz sand), factor D: rosin quality/(rosin and alcohol). Specific experimental factors and level settings are shown in Table 1.

Table 1 illustrates how varying these ratios affects the mechanical properties of the material, including elastic modulus (E), cohesion (c), and internal friction angle (φ).

The standard cylindrical molds (Φ50 mm × 100 mm) were used to prepare samples for triaxial compression tests, which measured elastic modulus, internal friction angle, and cohesion. The materials, including iron concentrate powder, barite powder, quartz sand, gypsum, rosin, and alcohol, were weighed and mixed thoroughly. The mixture was compacted into the molds in layers, and after demolding, the samples were subjected to triaxial testing to determine their mechanical properties.

### Triaxial Compression Tests

In a triaxial compression test, the stress–strain (σ-ε) curve of the specimen is obtained by strain loading. The shear rate of this test was set at 0.0125 mm/s. Taking the peak value of the curve as the failure point of similar materials, the cohesive force and internal friction angle of similar materials were obtained.

Triaxial compression tests were conducted on the 10 groups of specimens, successfully determining the cohesive force, internal friction angle, and elastic modulus for each group. The specific results are presented in Table 2. According to the test data, the elastic modulus of the similar materials ranged from 17,381.52 kPa to 74,944.15 kPa, while cohesion values varied from 24.09 kPa to 259.90 kPa, and the internal friction angle ranged from 17.10° to 46.28°. These findings reveal a wide range of mechanical parameters, demonstrating the flexibility and adaptability of the similar material ratio scheme used in this study. The significant variation in these mechanical properties indicates that the material configuration can effectively simulate the diverse mechanical behavior of heavily weathered granite found in the coastal regions of Fujian Province. This variability allows for more accurate replication of the geological conditions in the region, enhancing the reliability of the material models for geotechnical engineering applications in similar terrains.

## 3. Analysis of Range and Variance of Each Factor

**Figure 2:**
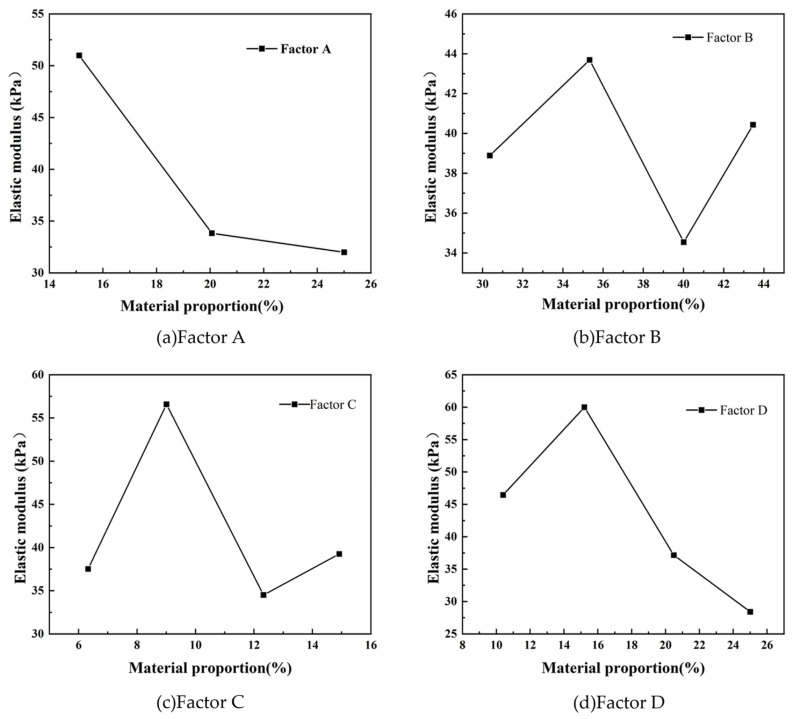
Range analysis of elastic modulus.

Range (R) is an important indicator used in statistics to measure the dispersion of data, which represents the difference between the maximum and minimum values in a data set. The larger the range value, the stronger the discreteness of the data. The smaller the range, the weaker the dispersion of the data. In this study, by drawing the range analysis diagram of the elastic modulus (see Figure 2), it can be clearly observed that the influence of various test factors on the elastic modulus of similar materials. According to the results of range analysis, the order of influence of each factor on elastic modulus is D > C > A > B. That is, factor D (binder concentration) has the greatest influence on the elastic modulus of similar materials, while factor A (iron concentrate powder content) and factor B (barite powder content) have relatively little influence on the elastic modulus. Further analysis of Figure 2 shows that the range of factor D first increases and then decreases with the change of ratio.

**Figure 3:**
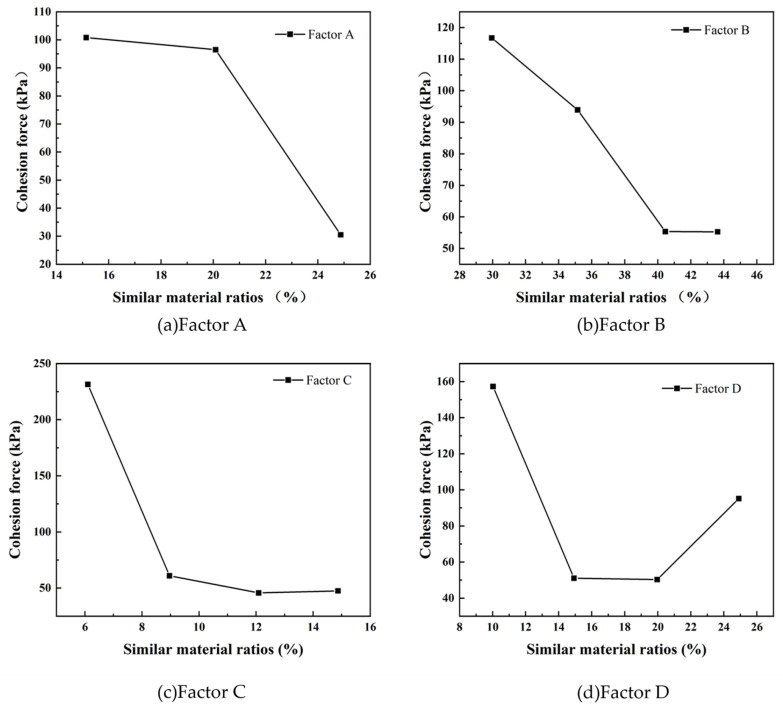
Cohesion range analysis.

As shown in Figure 3, the influence of each test factor on the cohesion of similar materials clearly follows the order C > D > A > B. This indicates that factor C (the content of rosin and gypsum) exerts the most significant impact on cohesion. As the content of gypsum and rosin increases, there is a noticeable decline in cohesion. In comparison, factor D (content of rosin) also shows a substantial effect on cohesion, but to a lesser extent than factor C. The effects of factor A (iron concentrate powder) and factor B (barite powder) are much weaker, with smaller variations in cohesion observed as their contents change. This also suggests that iron concentrate and barite powders contribute less significantly to the overall cohesion of the similar materials.

**Figure 4:**
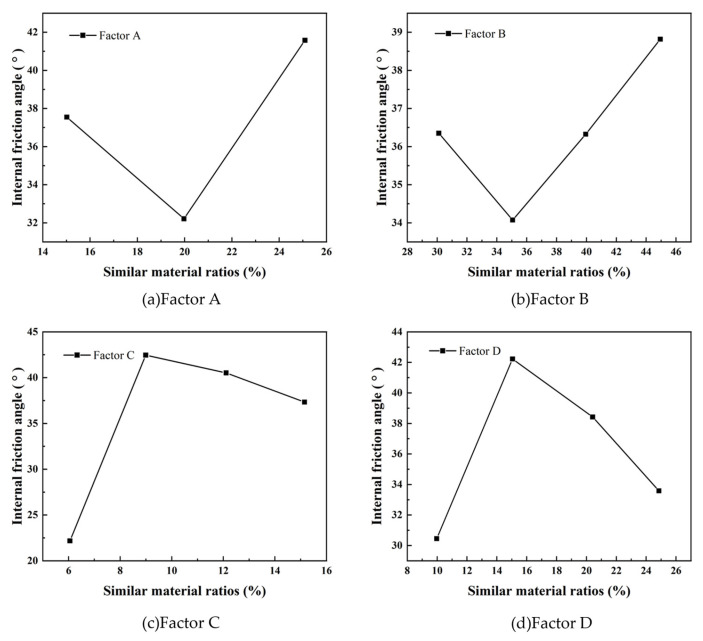
Range analysis of internal friction angle.

In Figure 4, the range analysis of the internal friction angle follows a similar trend to that observed in the cohesion results. The order of influence of the test factors on the internal friction angle is C > D > A > B, indicating that factor C (rosin and gypsum content) has the most significant impact. This is consistent with the cohesion analysis, where rosin and gypsum also had the greatest effect. In contrast, factor A (iron concentrate powder) and factor B (barite powder) have a negligible influence on the internal friction angle. A further look at Figure 4 reveals that the effect of factor C on the internal friction angle exhibits a non-linear behavior, initially increasing and then decreasing. This suggests that there is an optimal range of rosin and gypsum content that maximizes the influence of the internal friction angle, beyond which the influence of internal friction decreases.

Table 3 presents the ANOVA results for factors A, B, C, and D in relation to the elastic modulus, cohesion, and internal friction angle under the condition of confidence level α = 0.2. By comparing the calculated F-values with their respective critical values, we can determine the degree of influence each factor has on the mechanical properties. When the F-value exceeds the critical value, the factor is considered to have a significant impact. As shown in the data, factor D has the greatest influence on the elastic modulus, factor C significantly affects cohesion, and factor C also impacts the internal friction angle. These results correspond to the findings from the range analysis, confirming that factor C and D play key roles in determining the material properties.

## 4. Rock-Socketed Single Pile Model Test

In the above research, we conducted a systematic similar material ratio test for strongly weathered granite. The mechanical properties of these materials were systematically measured, with the elastic modulus ranging from 17,381.52 to 74,944.15 kPa, cohesion from 24.09 kPa to 259.90 kPa, and internal friction angle from 17.10 to 6.28°. Among the specimens tested, Specimen 1 exhibited mechanical properties most closely matching the heavily weathered granite in Fujian Province. As a result, Specimen 1 was selected as the foundation material for further testing of the bearing characteristics of rock-socketed single piles.

### 4.1. Design of Model Box and Pile

The single pile model was made of aluminum pipe. According to the actual size of large offshore wind power single pile, the similarity ratio CL = 1/50 model was designed. The corresponding pile diameter was 0.12 m, the pile length was 1.2 m, and the depth of the pile buried below the foundation surface was 0.8 m.

**Figure 5:**
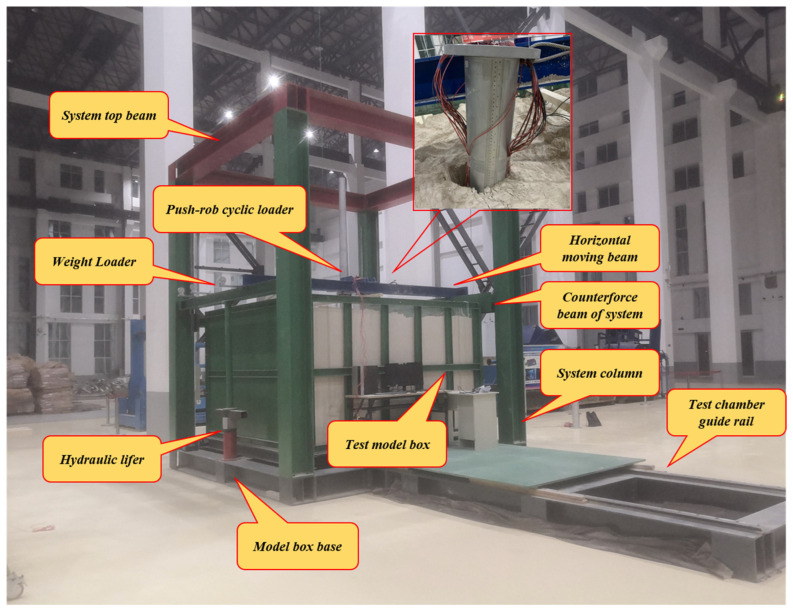
Model box and test environment.

As shown in Figure 5, a model box was welded through a steel frame. According to the relevant literature research, when the distance between a single pile and the inner wall of the model box is greater than three times the pile diameter, the influence of the boundary effect can be effectively eliminated. Therefore, the size of the model box designed in this experiment was 1.2 m × 1.2 m × 1.2 m to ensure the accuracy of the experimental results.

In the model test, the similar soil of Specimen 1 was selected to simulate the strongly weathered granite in the coastal area of Fujian Province. The specific ratio was: A factor 15%, B factor 30%, C factor 6%, D factor 10%. The soil mass was filled in layers and was compacted every 20 cm until the height of the foundation met the design requirements. The installation of the pile body adopted the embedded method. When the soil was filled to the specified height, the single pile was fixed to its design position, and then the soil was filled and compacted. After the pile–soil installation was completed, the specimen was placed in a natural state for 15 days to ensure that the soil characteristics were closer to the actual working conditions and provide more reliable experimental conditions for subsequent test studies.

### 4.2. Model Test Acquisition and Loading Device

**Figure 6:**
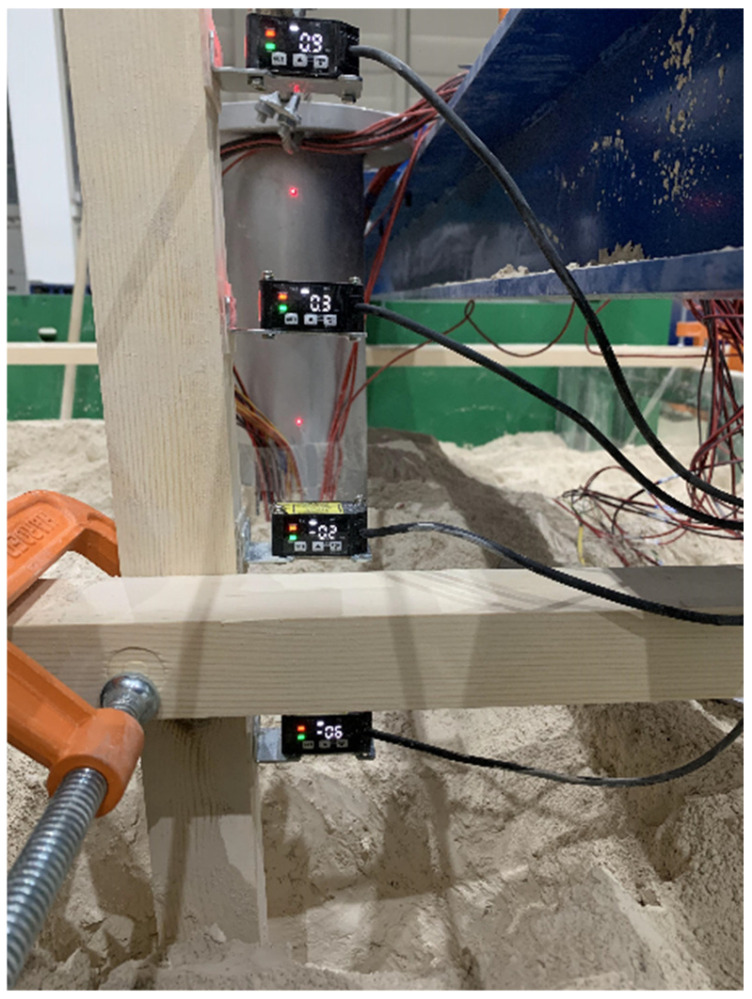
Laser displacement meters at different positions.

The displacement was measured by a laser displacement meter with a measuring range of 5 mm (Figure 6). The laser displacement meter was used to point laser beams at different positions of the piles to mark them. In this way, the displacement data of the piles above the ground were accurately measured.

Load sensors with a range of 50 kN were used for load measurement. Their working principle is based on the resistance strain effect: when a sensor is subjected to external force, the length of the internal resistance wire is shortened, which causes the resistance to decrease, and finally the load size is read by means of bridge assembly and deployment.

The loading device of the horizontal load adopted a loading system combining weights and pulleys. The system used weights of different masses at the bottom to generate vertical loads, and used a pulley system to convert vertical loads into horizontal loads. In order to study the displacement of pile top under different horizontal loads, weights of different masses were applied to pile top in the experiment. When the pile body reached the stable state, the horizontal displacement data of the pile top were accurately measured and recorded by the laser displacement meter. Each group of experiments was repeated three times, and the average value was taken as the final experimental result data.

The loading device for the vertical load adopted a jack stage loading system. A load sensor was arranged on the top of the model pile to record the vertical load exerted by the jack on the top of the pile.

**Figure 7:**
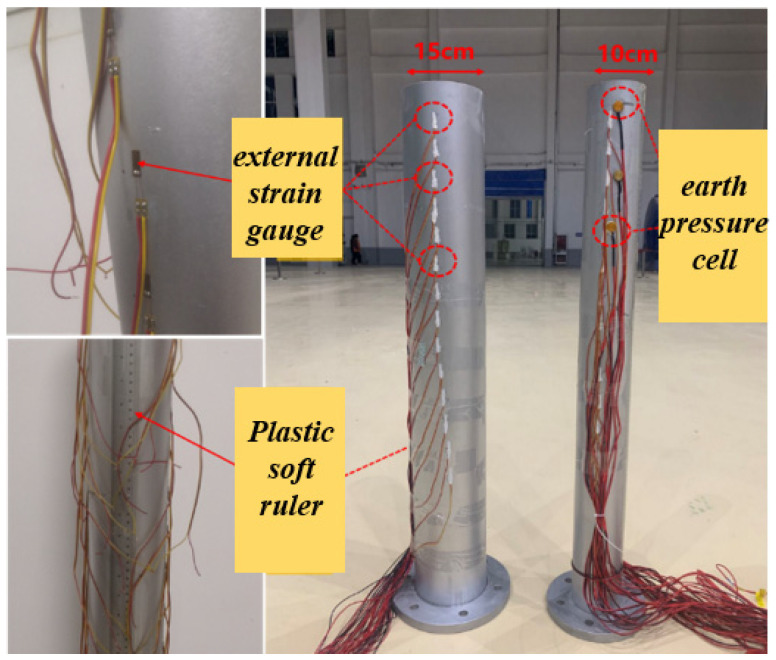
Strain gauge installed on model pile.

As shown in Figure 7, in order to obtain the bending moment of the pile body, strain gauges were symmetrically attached on both sides of the model pile. First, a cotton swab with or without ethanol and acetone was used to carefully wipe the strain gauge and its adhesive parts, and a special adhesive was used to firmly paste the strain gauge on the part to be measured. The wire was then welded to the terminal of the strain gauge and drawn out along the pile body. In order to avoid damage to the strain gauges and wires during the test, insulation tape was used to fix all strain gauges after installation.

### 4.3. Analysis of Rock-Socketed Single Pile Model Test Results

Through the data acquisition of model test and loading device, the variation law of load–displacement at the top of pile, the variation law of pile displacement above the ground, and the variation law of pile bending moment were analyzed.

**Figure 8:**
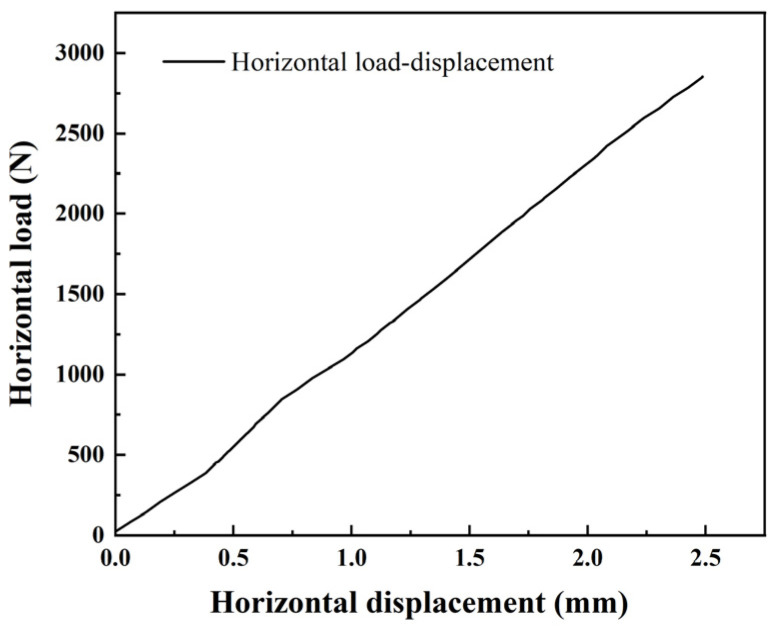
Horizontal load–displacement curve.

According to the laser displacement meter and the horizontal loading system, the horizontal load–displacement curve of the model pile was obtained, as shown in Figure 8. The curve demonstrates an approximately linear relationship between horizontal load and displacement; this linearity suggests that the pile did not experienced any significant plastic deformation or failure up to the maximum applied horizontal displacement of 2.5 mm.

**Figure 9:**
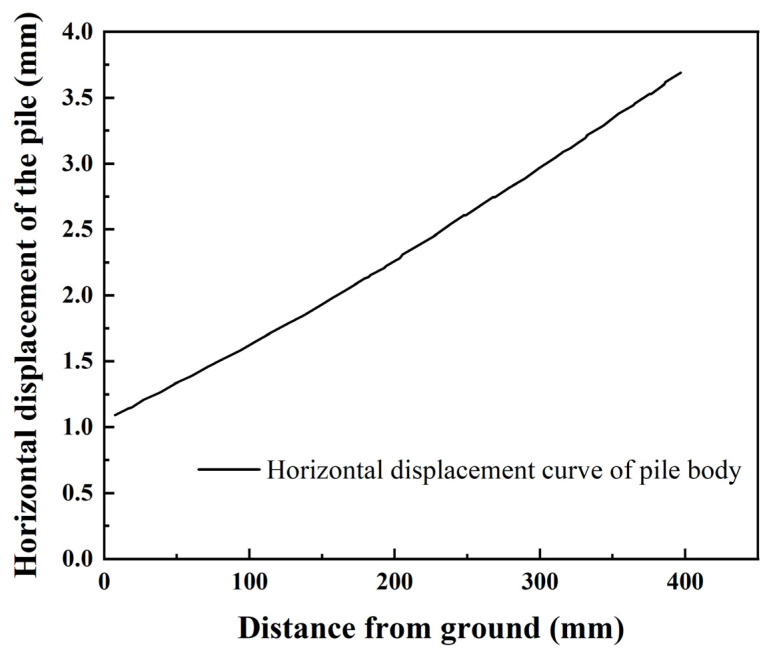
Horizontal displacement curve of pile body.

The displacement of the pile was read by the laser displacement meter set above the ground, and the deformation characteristics of the pile at different heights were analyzed. Figure 9 shows the horizontal displacement of the pile, with non-linear deformation near the ground and more linear behavior near the top. The non-linearity at the base due to greater soil resistance limits the displacement, while the linear portion at the top suggests the pile acts like a flexible cantilever. This behavior is characteristic of a flexible pile, with the upper portion experiencing more bending under horizontal loads while the lower portion remains constrained.

The tensile and compressive strains at different positions of the pile body were measured by the strain gauge arranged along the length of the pile to calculate the bending moment of the section. The specific calculation expression is as follows:

(1) $$M=\frac{\varepsilon EI}{R}$$

where M is the bending moment of the position to be measured, ε is the measured strain, E is the elastic modulus of the pile body, I is the moment of inertia of the section, and R is the radius of the model pile.

**Figure 10:**
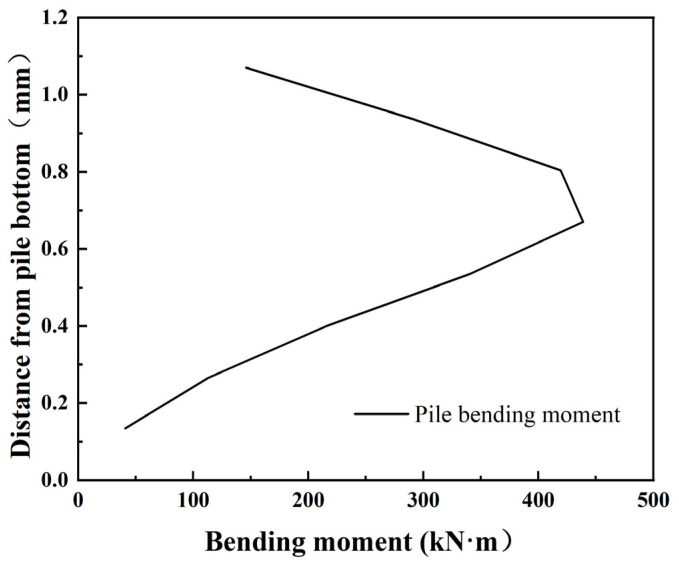
Bending moment curve with 1.1 mm top displacement.

A horizontal displacement of 1.1 mm was applied to the pile top by the horizontal loading system, and the bending moment change curve of the pile body was calculated according to the measured strain value and the above formula. In Figure 10, the bending moment distribution along the pile depth shows that it first increases and then decreases. The maximum bending moment occurs at 0.67 m from the pile bottom and is close to zero at the pile bottom. The reduction in bending moment as we approach the pile bottom suggests that the heavily weathered granite foundation still provides the support to limit further bending.

**Figure 11:**
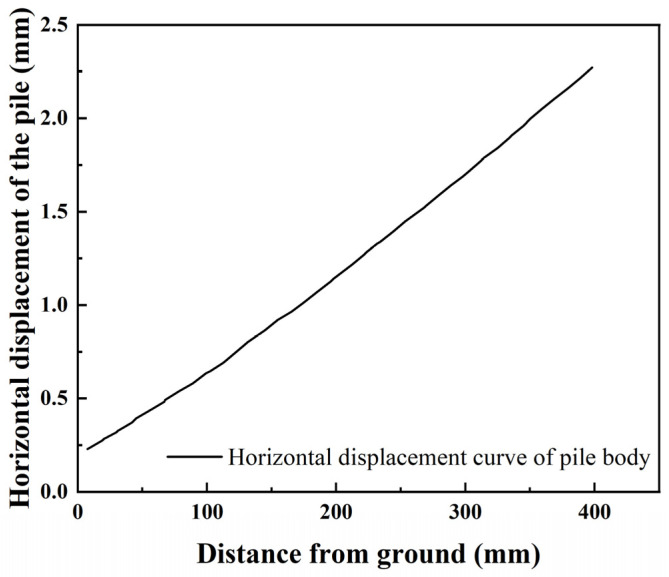
Horizontal displacement curve of pile with 2.3 mm top displacement.

**Figure 12:**
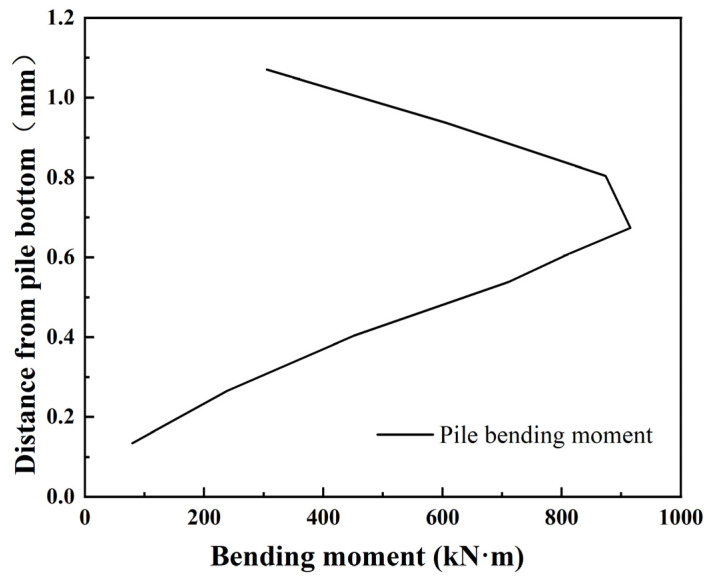
Bending moment curve with 2.3 mm top displacement.

As shown in Figure 11 and Figure 12 below, the combined system of weights and pulleys was used to continue to increase the displacement of pile top, and the deformation and bending moment of the pile body were analyzed when the horizontal displacement of pile top was 2.3 mm.

According to the above horizontal deformation characteristics of the pile body under different horizontal loads, it can be found that due to the imbedding effect of strongly weathered granite on pile body, the deformation of the pile body appears an inflection point near the ground; that is, the model piles all behave in the form of flexible piles under different horizontal loads. The horizontal deformation changes linearly with the increase of the distance between pile body and ground. When the horizontal displacement of the pile top reaches 2.3 mm, the pile slope is approximately 0.0045; when the horizontal displacement of the pile top reaches 3.7 mm, the pile slope is approximately 0.00725, an increase of 61%.

It can be seen from the analysis of pile bending moment when the model pile is subjected to different lateral forces that the bending moment of the pile body increases with the increase of pile top displacement. When the lateral displacement of pile top is 1.1 mm, the maximum bending moment of model pile is 439 N·m. When the displacement of pile top reaches 2.3 mm by increasing the horizontal load, the maximum bending moment appears at 0.67 m away from pile bottom, and the maximum bending moment is 916 N·m, which is 2.09 times of the original.

**Figure 13:**
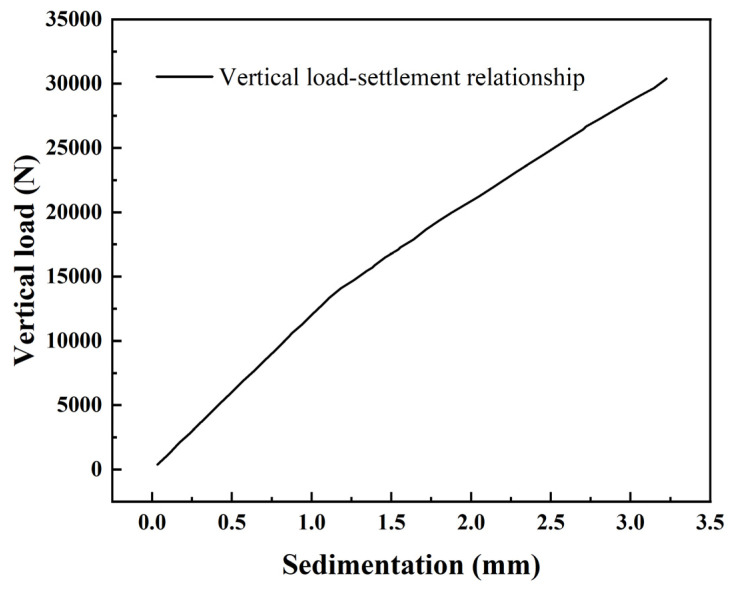
Vertical load–settlement curve.

The vertical load was applied to the pile top by using the dry weight top, the settlement amount of the pile top was read by the laser displacement meter, and the vertical load was read by the load sensor. The vertical load–settlement curve of the model pile is drawn as shown in Figure 13. It can be seen that under vertical load, there is an obvious inflection point in the load–settlement curve of pile top, which presents the characteristics of piecewise change. When the pile tip settlement is less than 1 mm, the vertical load–settlement curve changes linearly, indicating that the foundation is in the stage of elastic change. When the settlement amount is greater than 1 mm, the slope of the curve suddenly drops.

## 5. Conclusions

In order to study the bearing characteristics of rock-socketed single pile along the southeast coast of Fujian Province, this paper explores the bearing characteristics of rock-socketed single pile from two directions of similar material ratio test and single pile model test. The conclusions are as follows:

(1) Successfully developed the similar materials to simulate the mechanical properties of strongly weathered granite from Fujian Province using a mix of iron powder, barite powder, quartz sand, rosin, alcohol, and gypsum. The elastic modulus ranged from 17,381.52 kPa to 74,944.15 kPa, cohesion from 24.09 kPa to 259.90 kPa, and internal friction angle from 17.10° to 46.28°.
(2) Binder concentration had the most significant influence on the mechanical properties, while gypsum notably affected cohesion and internal friction. Iron and barite powders had minimal impact on the elastic modulus.
(3) Specimen 1 closely matched with the mechanical properties of strongly weathered granite in Fujian Province. And the rock-socketed single pile model test showed the pile remained elastic under small displacements, with the maximum bending moment at 0.67 m from the pile base, indicating strong resistance to rotational failure.

## 6. Prospect

While this study provides a valuable reference for designing pile foundations of offshore wind projects in weathered granite, there are several limitations. The study focused solely on one material composition (Specimen 1), leaving other compositions and geological conditions unexplored. Furthermore, the long-term performance of the pile foundations under cyclic or dynamic loading was not addressed. Future research could explore additional material ratios to simulate a broader range of geological conditions, investigate long-term performance under cyclic loading, conduct field tests to validate the laboratory findings, and develop more sustainable, economical materials for similar applications.

## Figures and Tables

**Table 1:** Proportions of similar materials.

Test No.	A/%	B/%	C/%	D/%
1	15	30	6	10
2	15	35	9	15
3	15	40	12	20
4	15	45	15	25
5	20	30	9	20
6	20	35	6	25
7	20	40	15	10
8	20	45	12	15
9	25	30	12	25
10	25	35	15	20

Factor A: quality of iron concentrate powder/(iron concentrate powder + barite powder + quartz sand), factor B: quality of barite powder/(iron concentrate powder + barite powder + quartz sand), factor C: (gypsum and rosin)/(iron concentrate powder + barite powder + quartz sand), factor D: rosin quality/(rosin and alcohol).

**Table 2:** Test results of similar material ratio.

Test No.	*E*/kPa	*c*/kPa	*φ*/°
1	52,950.31	259.90	26.65
2	74,944.15	40.19	46.28
3	33,544.21	50.80	38.55
4	43,296.86	51.88	38.89
5	38,391.74	65.21	38.63
6	17,381.52	210.60	17.10
7	34,992.41	59.21	34.11
8	43,775.24	57.47	38.77
9	24,380.39	24.09	43.91
10	39,874.83	32.54	38.93

**Table 3:** Analysis of variance of similar materials.

Factor		A	B	C	D
Elastic modulus	SS	785.06	145.18	713.89	1209.21
	DF	2	3	3	3
	MS	392.53	48.394	237.962	403.07
	F	1.89	0.14	0.94	2.35
	Critical value	2.043	2.113	2.113	2.113
	Significance	Not	Not	Not	Yes
Cohesion range	SS	8099.8	6863.7	56,384.3	17,842.5
	DF	2	3	3	3
	MS	4049.92	2287.89	18,794.8	5947.5
	F	0.56	0.26	44.54	0.87
	Critical value	2.043	2.113	2.113	2.113
	Significance	Not	Not	Yes	Not
Internal friction angle	SS	127.779	27.168	545.527	191.783
	DF	2	3	3	3
	MS	63.8894	9.056	181.842	63.9276
	F	0.85	0.09	10.04	0.83
	Critical value	2.043	2.113	2.113	2.113
	Significance	Not	Not	Yes	Not

SS: Sum of Squares, DF: Degree of Freedom, MS: Mean Square, F: F-statistic.

## Data Availability

The original contributions presented in this study are included in the article; further inquiries can be directed to the corresponding authors.
